# Transcriptomic Profiles of Brain Provide Insights into Molecular Mechanism of Feed Conversion Efficiency in Crucian Carp (*Carassius auratus*)

**DOI:** 10.3390/ijms19030858

**Published:** 2018-03-14

**Authors:** Meixia Pang, Weiwei Luo, Beide Fu, Xiaomu Yu, Ying Zhou, Jingou Tong

**Affiliations:** 1 State Key Laboratory of Freshwater Ecology and Biotechnology, Institute of Hydrobiology, The Chinese Academy of Sciences, Wuhan 430072, China; pang1mei2xia3@163.com (M.P.); weiweiluo66@163.com (W.L.); fubeide@163.com (B.F.); xmyu@ihb.ac.cn (X.Y.); Memoria_Y@163.com (Y.Z.); 2 University of Chinese Academy of Sciences, Beijing 100049, China

**Keywords:** crucian carp (*Carassius auratus*), feed conversion efficiency, brain, differentially expressed genes, RNA-Seq

## Abstract

Feed efficiency is an economically crucial trait for cultured animals, however, progress has been scarcely made in the genetic analyses of feed conversion efficiency (FCE) in fish because of the difficulties in measurement of trait phenotypes. In the present investigation, we present the first application of RNA sequencing (RNA-Seq) combined with differentially expressed genes (DEGs) analysis for identification of functional determinants related to FCE at the gene level in an aquaculture fish, crucian carp (*Carassius auratus*). Brain tissues of six crucian carp with extreme FCE performances were subjected to transcriptome analysis. A total of 544,612 unigenes with a mean size of 644.38 bp were obtained from Low- and High-FCE groups, and 246 DEGs that may be involved in FCE traits were identified in these two groups. qPCR confirmed that genes previously identified as up- or down-regulated by RNA-Seq were effectively up- or down-regulated under the studied conditions. Thirteen key genes, whose functions are associated with metabolism (*Dgkk*, *Mgst3* and *Guk1b*), signal transduction (*Vdnccsa1b*, *Tgf*α, *Nr4a1* and *Tacr2*) and growth (*Endog*, *Crebrtc2*, *Myh7*, *Myh1,* *Myh14* and *Igfbp7*) were identified according to GO (Gene Ontology) and KEGG (Kyoto Encyclopedia of Genes and Genomes) annotations. Our novel findings provide useful pathway information and candidate genes for future studies of genetic mechanisms underlying FCE in crucian carp.

## 1. Introduction

Aquaculture products are among the most important sources of nutrition for people around the world [1]. Fish is a key source of protein, essential amino-acids and minerals, especially in low-income, food-deficient countries [2,3,4]. In the past decades, yield-related traits in fish have been extensively studied by researchers to meet the ever-increasing global demand and increase financial returns [5,6]. Since feed cost comprises about 65–75% of the total production cost in most aquaculture species [7], the increasing production demand with huge input cost kept up pressures on us to investigate how to improve feed efficiency. In this sense, breeding fish with high feed efficiency would mean big savings and enhance the profitability of producers.

Feed efficiency was usually measured as feed conversion ratio (FCR), which is the ratio of feed intake to body weight gain [8]. To date, some DNA variants playing roles in feed efficiency have been proposed in livestock [8,9,10,11] and poultry industry [12,13,14,15] using quantitative trait loci (QTL) mapping and association studies. Those identified candidate genes pertain to numerous biological processes [10,16], suggesting that biological strategies involving feed efficiency are diverse. RNA sequencing (RNA-Seq) technology combined with the analysis of differentially expressed genes (DEGs) are reliable and precise ways to provide useful information for molecular mechanism of complex characters [17]. Moreover, RNA-Seq has attracted considerable interests and received great success concerning many economic traits in some other cultured species [18,19,20]. An increasing number of studies have addressed the transcriptomic profiles of tissues and organs to examine the variety of functional pathways underlying inter-individual difference in feed efficiency [21,22,23,24,25], which focused on target organs such as intestine [21] and liver [22,23], but no similar analysis has been done in neural organs. Moreover, genetic studies on feed efficiency have been scarcely reported in aquaculture species [26,27].

Crucian carp (*Carassius auratus*) is an economically important aquaculture species worldwide [28]. For this fish, progress on improving feed efficiency has mainly focused on changes in external conditions [29,30]. To date, the only one study about OTL mapping for feed conversion efficiency (FCE, the inverse of FCR) was reported by our laboratory in crucian carp [31]. In a previous study, seven candidate genes associating with FCE and its relevant traits were identified, such as *mapk11*, *cse1l*, *fam126b*, *myh14*, *rgs9bp*, *cldn10a* and *cldn10b*, involved in several biological functions. So far, few transcriptome analyses have been conducted in crucian carp. Liao, et al. conducted differential gene expression analyses among four tissues [32], and Li, et al. performed RNA-Seq to determine functional differences and DEGs between gibel carps and crucian carp using ten tissues of each species [33]. No transcriptome analysis has been conducted for FCE in this fish. Here, we used high-throughput sequencing of mRNAs from brain tissues of six crucian carps with extreme phenotypes of FCE. We aimed to find novel genes and biological pathways that may be related to feed efficiency of crucian carp, which would provide a better understanding of genetic mechanisms underlying FCE in fish.

## 2. Results

### 2.1. Transcriptome Sequencing and Statistics of Unigenes

After quality filtering, the RNA-Seq of six brain samples yielded around 52.59 million high-quality sequence data. The Q30 values of each sample were up to 93.20%, and GC-content of each sample ranged from 45.33% to 49.44% (Table 1). The clean reads obtained from the six transcriptome libraries were assembled to full-length transcripts, and a total of 544,612 unigenes were achieved after elimination of redundant transcripts. Summary data of the assembled transcripts and unigenes are given in Table 2, and the number of unigenes per size that normally decreases continuously is shown in Table 2 and Figure S1.

### 2.2. Functional Annotation and Classification of Unigenes

All the unigene sequences were subjected to a search against Nr [34], Swissprot [35], COG [36] and KEGG [37] databases. Among these unigenes, totally 76,060 unigenes (13.97% of all the 544,612 unigene sequences) were annotated in four public databases, including 68,168 (12.52%) in Nr, 27,535 (5.06%) in Swissprot, 12,550 (2.30%) in COG and 29,390 (5.40%) in KEGG, respectively.

**Figure 1:**
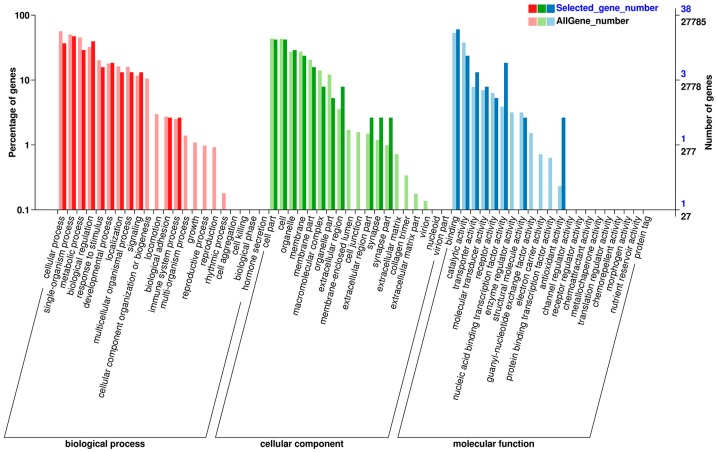
GO classifications of all unigenes and DEGs associating with feed conversion efficiency from brain samples of crucian carp.

GO assignment was performed to classify functions of the predicted crucian carp genes. Based on sequence homology, a total of 27,785 unigenes were annotated to 62 terms of GO classification, which were classified into three major functional categories: biological process (21,797 unigenes, 78.45%), cellular component (16,807 unigenes, 60.49%) and molecular function (22,584 unigenes, 81.28%) (Figure 1).

### 2.3. Identification of Differentially Expressed Genes (DEGs)

Overall, 61.15% ± 3.26% of clean reads per sample were mapped back to the assembled transcripts (Table 1); 246 DEGs (Table S1) were identified in the comparison of Low_vs_High FCE groups, with 132 up-regulated and 114 down-regulated genes in the High group, respectively. Among these DEGs, a total of 113 genes were annotated in at least one of the four (Nr, Swissprot, COG and KEGG) databases.

### 2.4. Enrichment for Functional Analysis of DEGs

**Figure 2:**
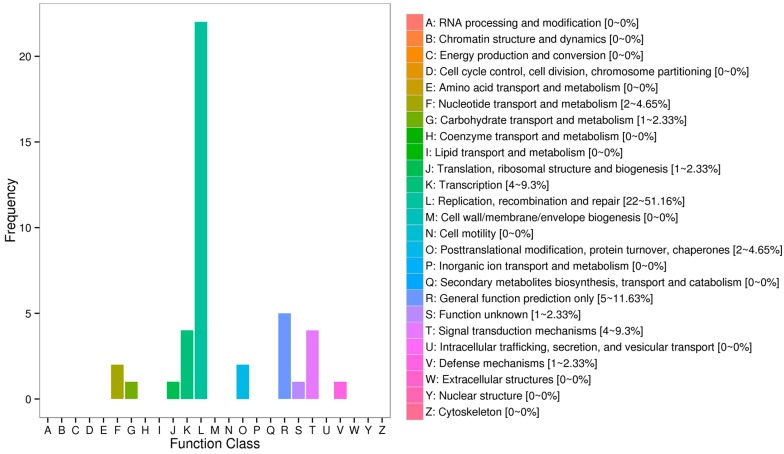
COG classifications of DEGs associating with feed conversion efficiency from brain samples of crucian carp.

The results of COG enrichment analysis for the annotated DEGs are shown in Figure 2. Replication, recombination and repair (L, 22–51.16%) annotated the most DEGs in the comparison of Low_vs_High FCE groups, followed by transcription (K, 4–9.3%) and signal transduction mechanisms (T, 4–9.3%).

A total of 38 DEGs were annotated to 30 terms of GO classification (Figure 1). Under the biological process category, cellular process (14 DEGs, 36.84%), single-organism process (18 DEGs, 47.37%), biological regulation (15 DEGs, 39.47%) and metabolic process (11 DEGs, 28.95%) have the most abundant GO function items. In the cellular component category, a significant percentage of genes were clustered into cell (16 DEGs, 42.11%), cell part (16 DEGs, 42.11%), organelle (11 DEGs, 28.95%) and membrane (nine DEGs, 23.68%). Within the molecular function category, most genes were assigned to binding (23 DEGs, 60.53%) and catalytic activity (nine DEGs, 23.68%).

Pathway analyses were performed using mapped DEGs to other species’ orthologues to get more insights into the effects of FCE on metabolisms in the crucian carp. 20 statistically enriched pathways (*p* < 0.05) were revealed by KEGG pathway analysis, including four in cellular processes, seven in environmental information processing, three in genetic information processing, six in metabolism, and two in organismal systems categories, respectively (Table 3).

### 2.5. Critical DEGs Involved in Feed Conversion Efficiency of Crucian Carp 

Among all DEGs identified by RNA-Seq analysis, most of the annotated DEGs were classified into molecular function associating with ATP/GTP binding (GO:0005524/GO:0005525), ATP catabolic process (GO:0006200), signal transduction (GO:0007165) and metabolic process (GO:0008152), suggesting that FCE in crucian carp was mainly associated by such kind of pathways as energy metabolism and signal transduction. KEGG analysis also classified DEGs mainly into metabolism and signal transduction, suggesting that these genes may be considered as key candidate genes associating with FCE. Three candidate genes were enriched in metabolism pathways, including *Dgkk* (Diacylglycerol kinase kappa), *Mgst3* (Microsomal glutathione *S*-transferase 3) and *Guk1b* (Guanylate kinase 1b). Whereas five genes were classified into signal transduction pathways, such as *Vdnccsa1b* (Voltage-dependent N-type calcium channel subunit alpha-1B-like) and *Tacr2* (Tachykinin receptor 2) in calcium signaling pathway, *Tgfα* (Transforming growth factor alpha) in ErbB signaling pathway, *Vdnccsa1b* and *Nr4a1* (Nuclear receptor subfamily 4 group A member 1) in MAPK signaling pathway, and *Dgkk* in phosphatidylinositol signaling system. Attentions to the DEGs with functions involved in growth, such as *Endog* (Endonuclease G), *Crebrtc2* (cAMP-response element binding protein CREB-regulated transcription coactivator 2-like), *Myh7* (myosin heavy chain 7), *Myh1* (Myosin heavy chain 1), *Myh14* (Myosin heavy chain 14) and *Igfbp7* (Insulin-like growth factor-binding protein 7), should be also paid because this trait is highly correlated with FCE.

### 2.6. Validation of RNA-Seq Results by Quantitative Real-Time RT-PCR (qRT-PCR)

**Figure 3:**
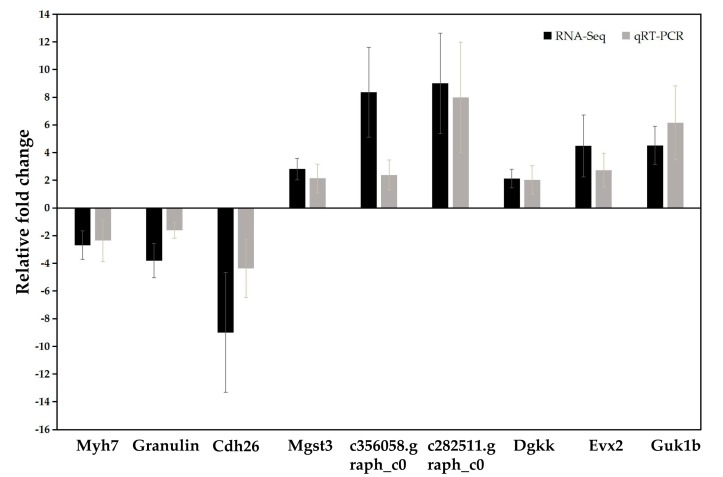
Illustration of qRT-PCR confirmation results for 9 randomly selected DEGs.

qRT-PCR was performed for nine randomly selected DEGs to validate the RNA-seq results obtained in the current study. These tested DEGs included c353179.graph_c0 (*Myh7*), c370016.graph_c0 (*Granulin*), c381143.graph_c0 (*Cdh26*), c359802.graph_c0 (*Mgst3*), c356058.graph_c0 (unnamed), c282511.graph_c0 (unnamed), c417566.graph_c1 (*Dgkk*), c385041.graph_c0 (*Evx2*) and c406285.graph_c0 (*Guk1b*). The fold changes detected by qRT-PCR were comparable with the RNA-Seq expression profiles. In general, the expression patterns obtained by qRT-PCR were similar to those revealed by RNA-seq (Figure 3), confirming the data reliability of up or down- regulated DEGs obtained by RNA-seq.

## 3. Discussion

Efficient production of animals plays an important role in livestock [38], poultry [21] and aquaculture [39] industries. This implies the reduction of input costs and the increase of financial returns for producers. In almost all animal production systems, feed cost is the major component (30–70%) of the total costs [40]. Improvement in feed efficiency means reducing the amount of feed resources needed to produce meat and contributing to environmental sustainability [41]. Moreover, it has been reported that feed efficiency is involved in numerous biological processes and functional pathways [10,16], and this trait can be improved by breeding and feeding strategies [24]. Eight QTL associated with FCE, an assessment criterion for feed efficiency, were mapped to four linkage groups, explaining 15.2–20.9% of the phenotypic variations, and seven candidate genes related to FCE and its relevant traits involved in several biological processes were identified in crucian carp in a previous study [34]. In this study, we performed a pioneering and comparative transcriptome analysis based on brain tissues of six crucian carp with extreme FCE phenotypes to help us to understand the molecular mechanisms of FCE. This study is, to the best of our knowledge, the first report of identification of functional determinants involved in FCE at the gene level by transcriptome analysis in fish.

The current RNA-Seq work provided high-quality sequences, and the proportions of mapped reads per sample ranged from 57.73% to 65.35% (Table 1), which ensured the accuracy and reliability of subsequent differential expression analysis. Feed efficiency could be influenced by feed intake to a large content [42], and the brain is a major part of the neural organs that regulates feed intake activity and metabolism in earlier stage of the whole physiological processes in fish. Therefore, fish with significantly different FCE traits may be reflected by RNA profiles in brain. In this study, a total of 544,612 unigenes with a mean size of 644.38 bp (Table 2) were obtained from two groups with extreme FCE performance (namely Low and High groups) of brain tissues in crucian carp, and 246 DEGs (Table S1) that may be involved in FCE were identified. Zhao, et al. identified 300 significantly differentially expressed transcripts in liver of pig [22], while Yi, et al. found 41 promising candidate genes in duodenal of chicken [21]. Compared to these similar reports conducted in other animals, moderate numbers of DEGs were found from brains of crucian carp in this investigation.

According to the results of our previous work conducted using QTL mapping methods [31], identified candidate genes associating with FCE could be classified into three categories: genes encoding GTPase/ATP binding protein, tight junction protein and signal transduction regulator. Moreover, the significantly enriched GO terms of all the predicted crucian carp DEGs in the brain identified in this study were mainly associated with molecular function related to ATP/GTP binding (GO:0005524/GO:0005525), ATP catabolic process (GO:0006200), signal transduction (GO:0007165) and metabolic process (GO:0008152), supporting the hypothesis that FCE of crucian carp are mainly regulated by such pathways as energy metabolism and signal transduction. It has also been reported that energy and vitamin A metabolism pathways in the liver were important for feed efficiency in pigs [22]. Due to relatively a small number of DEGs being annotated in curcian carp, only three candidate genes (*Dgkk*, *Mgst3* and *Guk1b*) were enriched in KEGG pathways of metabolism, including lipid metabolism, glutathione metabolism, purine metabolism, xenobiotics biodegradation and metabolism that are involved in immune system (Table 3). *Dgkk* is a member of *Dgk* (Diacylglycerol kinase) family, which plays an important role in modulating the balance between diacylglycerol and phosphatidic acid [43]. *Mgst3* is related to defense mechanisms associated with oxidative stress by utilizing reduced glutathione [44]. For *Guk1b*, it can affect the metabolism of GTP [45]. A lipid metabolism pathway was the mostly reported pathway that may associate with feed efficiency in other animals [21,24,25,46], suggesting that *Dgkk* may be considered as an especially critical candidate gene affecting FCE in crucian carp. Moreover, genes related to the immune system were also identified in the transcriptome analyses of feed efficiency in beef [23] and pigs [24].

Another large class of concerned pathways involved in FCE is signal transduction. In this study, calcium signaling pathway (*Vdnccsa1b* and *Tacr2*), ErbB signaling pathway (*Tgf*α), MAPK signaling pathway (*Vdnccsa1b* and *Nr4a1*) and phosphatidylinositol signaling system (*Dgkk*) were found (Table 3) from brain tissues of crucian carp. Among these pathways, ErbB signaling could regulate cell proliferation, differentiation and apoptosis through Akt, MAPK and other signaling pathways. Genes enriched in MAPK signaling pathway related to feed efficiency were generally reported by previous researchers [46,47], such as *Fgf* (Fibroblast growth factor), *Tgfbr* (Transforming growth factor beta receptor), *Pkc* (Protein kinase C) and *Hsp72* (72-kd heat shock protein) in beef cattle. Additionally, similar candidate genes associating with FCE have also been found in other animals, which suggested that techniques and strategies employed in these studies were reliable and feasible. *Tgfα* is an up-regulating gene in High group of crucian carp involved in ErbB signaling pathway in this study, while *Tgfbr* is also associated with feed efficiency in chicken [48] and common carp [26]. *Igf1* (Insulin-like growth factor 1) is an important regulator of muscle growth and energy metabolism [49,50] that was identified as a candidate gene involved in feed efficiency in chicken [51] and common carp [26]. *Igfbp3* (Insulin-like growth factor-binding protein 3) and *Igfbp7* are *Igf* binding proteins in circulation and played important roles in modulating *Igf* bioavailability and half-life, which were respectively found in beef [23] and crucian carp. Both *Myh7* and *Myh1* were digged out in this report that may be involved in regulating muscle growth, and *Myh7* has also been found to be differentially expressed between low- and high-FCR groups in pigs [22]. Moreover, *Myh14,* another gene from *Myh* superfamily that plays an important role in fish muscle formation [52], has been identified in our previous work conducted using QTL mapping method in crucian carp [31]. The consistent result obtained in both studies suggesting that *Myh14* may be also considered as an especially critical candidate gene affecting FCE in crucian carp.

The reliability of RNA-Seq analysis was confirmed by the concordance between the computational and experimental results by employing nine randomly selected DEGs for qRT-PCR assays in this study, which was similar to some previous studies in animals [53,54,55]. Since there is no reference genome for *Carassius auratus*, most unigenes were not well annotated in all annotation databases. At present, a well-assembled genome of crucian carp is needed, and it may help to find more key candidate genes potentially related to feed efficiency. Our results would provide useful information for a physiological basis to develop improved feed formulas and/or feeding conversions. Furthermore, these novel findings would be useful for such future studies as association analysis and functional verification between candidate genes and FCE traits, thereafter providing proper markers for marker-assisted selection for potential improvement of feed efficiency in crucian carp.

## 4. Materials and Methods

### 4.1. Sample Collection and RNA Preparation

A reference family consisting of 120 diploid crucian carp fingerlings, generated by parents from Zhangdu Lake, Yangtze River (Wuhan, China) using artificial crossing in April, 2015, was used in this study. At 82 days post hatch (dph), these reference fish (mean body weight of 0.87 ± 0.39 g) were individually reared in the same environment with pallet feed in a series of re-circulating aquarium tanks in order to achieve accurate feed consumption. Such detailed conditions of the aquarium tanks as water temperature (27–28 °C), dissolved oxygen (7–8 mg/L) and water flow rate (1 ms^−1^), were regularly maintained throughout the feeding test for two months. During the experimental period, all test fish were fed three times (10:00 am, 15:00 pm and 20:00 pm) a day by the same fish-feeder, and the fish-feeder stopped feeding when the fish satiated each meal. Faeces generated by experimental fish in each tank were siphoned out once a day and a complete water change was made once a week. The individual body weight (BW) was recorded at the start (initial BW, BW_I_) and the end (final BW, BW_F_) of the experiment to calculate BW gain. The feed conversion efficiency (FCE) was estimated with the model as follows:

(1) $$\mathrm{FCE}=(BW_{F}-BW_{I})/\mathrm{FI}$$

Here FCE = feed conversion efficiency, BW_F_ = final body weight, BW_I_ = initial body weight, FI = total feed intake of each individual, which was recorded as the difference between the final and the beginning weight of diet used during the test. The detailed information of this feeding test was described in previous study [31].

Fish with extreme FCE phenotypes were used for RNA extraction. At the end of the whole experimental period, we selected six fish consisting of two groups (three biological replicates per group) to represent two divergent FCE performances (Low group: L1–L3 and High group: H1–H3). Table 4 detailed the measurements of FCE and its relevant traits in two groups. For RNA isolation, brain tissues were sampled immediately from sacrificed fish, frozen in liquid nitrogen and then stored at −80 °C until further processing. All experimental procedures involved in fish in this study were based on institutional regulations and guideline for experimental animals of the Hubei Provincial Committee for Animal Welfare (Permit Number: 20130522-02).

### 4.2. RNA Extraction, Library Preparation and Transcriptome Sequencing

Total RNA was isolated from the frozen brain samples using Trizol Reagent (Invitrogen, Carlsbad, CA, USA) according to the manufacturer’s instructions. The RNA degradation and contamination was monitored on 1% agarose gels. RNA purity and concentration was measured by NanoPhotometer^®^ spectrophotometer (IMPLEN, Westlake Village, CA, USA) and Qubit^®^2.0 Flurometer (Life Technologies, Carlsbad, CA, USA). The RNA integrity number was assessed using the RNA Nano 6000 Assay Kit of the Agilent Bioanalyzer 2100 system (Agilent Technologies, Santa Clara, CA, USA), which ranged from 8.2 to 8.9, suggesting that the samples were well preserved to meet the cDNA library construction requirements.

Sequencing libraries were generated using NEBNext^®^Ultra™ RNA Library Prep Kit for Illumina^®^ (NEB, Ipswich, MA, USA) according to manufacturer’s recommendations. Oligo-dT beads (Qiagen, Dusseldorf, Germany) were used to separate poly (A) mRNA from the total RNA of each sample, and the fragmentation buffer was added to split all mRNA into short fragments. Then the first-strand cDNA was synthesized using the random hexamer-primed reverse transcription, and the second-strand cDNA was generated using RNase and DNA polymerase I. The cDNA fragments were washed by EB buffer for end reparation poly (A) after purification, and index codes were added to attribute sequences to each sample. Fragments with suitable size for sequencing were isolated from the agarose gels, and PCR amplification was used to enrich these fragments to construct the final cDNA libraries. Finally, the cDNA libraries were sequenced on an Illumina Hiseq 2000 platform and paired-end reads were generated.

### 4.3. Transcriptome Assembly and Functional Annotation

For ensuring high-quality data, raw data (raw reads) of fastq format were firstly processed through in-house Perl scripts, which eliminated all those reads with sequencing adapter and nucleotides in reads with quality value less than 20 in both end. In this step, good quality sequences of clean data (clean reads) were obtained by abandoning reads containing adapter, reads containing ploy-N and low-quality reads from raw data. At the same time, Q30, GC-content and sequence duplication level of the clean data were calculated. The clean data of this article are publicly available in the NCBI Sequence Reads Archive (SRA) with accession number PRJNA433432.

All clean reads of the six libraries were jointly assembled into contigs employed by Trinity software [56]. Since there is no reference genome of *Carassius auratus*, a k-mer value cutoff of 25 was used after removing redundant nucleotide sequences by Tgicl (v2.1, http://sourceforge.net/projects/tgicl/files/tgicl%20v2.1/). Then, unigenes were generated by connecting the contigs (longer than 200 bases) to obtain sequences that could not be extended on either end, and maximum length non-redundant unigenes were acquired by further splicing and assembling using TGICL clustering software (J. Craig Venter Institute, Rockville, MD, USA). Finally, unigenes were aligned against the Nr (NCBI non-redundant protein sequences), Swissprot (A manually annotated and reviewed protein sequence database), COG (Clusters of Orthologous Groups of proteins), and KEGG (Kyoto Encyclopedia of Genes and Genomes) of protein databases using BlastX with an *E*-value <10^−5^. GO (Gene Ontology) annotation of these unigenes was performed using Blast2GO (https://www.blast2go.com/) based on the results of the NCBI Nr database annotation. Blastn was used to align these unigenes with the Nr database to search for proteins with the highest sequence similarity to the given unigenes and annotate their protein functions at the same time.

### 4.4. Analysis of Differentially Expressed Genes (DEGs)

Gene expression levels were estimated by RSEM (RNA-Seq by Expectation Maximization) [57] software package for each sample. The mapped reads were normalized according to fragment per kilobase of exon model per million mapped reads (FPKM) for each unigene between the two groups (Low_vs_High). Differentially expressed genes (DEGs) between the two groups were identified by the DEGseq package (samples with three biological replicates) applying the MA-plot-based method with Random Sampling model (MARS) method. In this study, DEGs with significant expression abundance between the two groups were selected using the following filter criteria: *p*-value < 0.01 and the absolute value of log2 Ratio ≥ 1, meaning each DEG between two groups should be at least two-fold. In order to determine the potential functions and metabolic pathways of these DEGs, COG, GO and KEGG enrichments were further analyzed. COG annotation of the DEGs was performed using Blastall software. GO enrichment analysis (*p*-value ≤ 0.05) of the DEGs was implemented by the topGO R packages based Kolmogorov–Smirnov test. Based on the hyper-geometric distribution model, we used KOBAS software [58] to test the statistical enrichment of DEGs in KEGG pathways, and the enrichment p-values were adjusted using the Benjamin and Hochberg method.

### 4.5. Validation of RNA-Seq Results by qRT-PCR

To confirm our differential expression results of RNA-Seq, we conducted quantitative reverse transcription PCR (qRT-PCR) assays for nine randomly selected DEGs in the same RNA samples used for RNA-Seq. First-strand cDNA was reverse-transcribed from total RNA using Reverse Transcriptase M-MLV (TaKaRa, Tokyo, Japan) with oligo-dT primer following the manufacturer’s instructions. The first-strand cDNA from each sample was diluted by 1:5 with sterile distilled water and used as template. Primers (Table S2) for qRT-PCR analyses of the nine genes were designed using Primer 5 Software, and qRT-PCR was performed on a StepOneTM Real-Time PCR System (Applied Biosystems, Foster City, CA, USA). The qRT-PCR reaction solution consisted of 6.5 μL Power SYBR Green PCR Master Mix (Applied Biosystems, Foster City, CA, USA), 0.2 μM of each forward and reverse primer, 1.2 μL diluted cDNA and 4.5 μL sterile distilled water. PCR program was 95 °C for 10 min, followed by 40 cycles of 95 °C for 15 s, 60 °C for 30 s, 72 °C for 45 s. Three parallel experiments were conducted in each run for each sample. The relative expression levels were normalized towards the internal control gene of β-actin. Optimized comparative Ct value (2^−ΔΔCT^) method [59] was used here to estimate gene expression levels.

## 5. Conclusions

We have reported a cerebric transcriptome of crucian carp using six fish with extremely low and high FCE. A total of 544,612 unigenes with a mean size of 644.38 bp were obtained from two FCE groups, and 246 DEGs that may be involved in FCE were identified. Based on GO and KEGG annotations, 13 DEGs related to metabolism, signal transduction and growth were identified to be key candidate genes associated with FCE traits. Our results provide valuable information for elucidating molecular mechanisms of feed conversion efficiency in fish, and these novel findings would be useful for such future studies as association analysis and functional genomics verification of candidate genes related to FCE in crucian carp.

## Figures and Tables

**Table 1:** Overview of the sequencing reads.

Samples	H1	H2	H3	L1	L2	L3
Raw reads	51,467,792	50,558,463	54,265,382	53,444,150	56,248,579	55,784,163
Clean reads	50,530,680	49,250,952	53,769,034	52,491,591	55,011,113	54,460,392
Q30	93.20%	93.76%	93.65%	93.37%	93.84%	93.79%
GC-content	48.04%	46.68%	48.84%	49.44%	47.37%	45.33%
Mapped reads	29,173,543	31,871,361	31,789,018	30,732,102	33,802,037	35,592,419
Mapped ratio	57.73%	64.71%	59.12%	58.55%	61.45%	65.35%

**Table 2:** Summary of the assembled transcripts and unigenes.

Length Range	Transcript	Unigene
200–300	283,078 (18.32%)	209,625 (38.49%)
300–500	264,160 (17.09%)	151,108 (27.75%)
500–1000	316,468 (20.48%)	102,120 (18.75%)
1000–2000	360,982 (23.36%)	50,873 (9.34%)
2000+	320,919 (20.76%)	30,886 (5.67%)
Total Number	1,545,607	544,612
Total Length	1,940,292,597	350,936,744
N50 Length	2091	965
Mean Length	1,255.36	644.38

**Table 3:** Pathways associating with feed conversion efficiency of crucian carp.

KEGG Category		Pathway Name	Pathway ID	DEGs
Cellular Processes	Cell growth and death	Apoptosis	ko04210	*Endog*
		Cell cycle	ko04110	*Crebrtc2*
	Cell motility	Regulation of actin cytoskeleton	ko04810	*Nckap1*
	Cellular community	Tight junction	ko04530	*Myh7*, *Myh*
Environmental Information Processing	Membrane transport	ABC transporters	ko02010	*Abcb11*
	Signal transduction	Calcium signaling pathway	ko04020	*Vdnccsa1b*, *Tacr2*, *Htr7*
		ErbB signaling pathway	ko04012	*Tgfα*
		MAPK signaling pathway	ko04010	*Vdnccsa1b*, *Nr4a1*
		Phosphatidylinositol signaling system	ko04070	*Dgkk*
	Signaling molecules and interaction	Cytokine-cytokine receptor interaction	ko04060	*Xcr1*
		Neuroactive ligand-receptor interaction	ko04080	*Tacr2*, *Htr7*
Genetic Information Processing	Replication and repair	Base excision repair	ko03410	*Cdcpcec1*
	Transcription	Basal transcription factors	ko03022	*Qtf2f2b*
	Translation	RNA transport	ko03013	*Tef1*
Metabolism	Lipid metabolism	Glycerolipid metabolism	ko00561	*Dgkk*
		Glycerophospholipid metabolism	ko00564	*Dgkk*
	Metabolism of other amino acids	Glutathione metabolism	ko00480	*Mgst 3*
	Nucleotide metabolism	Purine metabolism	ko00230	*Guk1b*
	Xenobiotics biodegradation and metabolism	Drug metabolism—cytochrome P450	ko00982	*Mgst 3*
		Metabolism of xenobiotics by cytochrome P450	ko00980	*Mgst 3*
Organismal Systems	Circulatory system	Adrenergic signaling in cardiomyocytes	ko04261	*Myh7*
		Cardiac muscle contraction	ko04260	*Myh7*

**Table 4:** Descriptive statistics of feed conversion efficiency and relevant traits in crucian carp.

Trait	Low Group	High Group
BW_I_ (g)	0.69 ± 0.12	0.78 ± 0.35
BW_F_ (g)	1.93 ± 0.37	4.25 ± 1.87
FI (g)	3.32 ± 0.59	4.63 ± 2.20
FCE	37.0 ± 4.1%	76.0 ± 3.0%

BW_I_: initial body weight, FI: total feed intake, BW_F_: final body weight, FCE: feed conversion efficiency.

## Supplementary Materials

Supplementary materials can be found at http://www.mdpi.com/1422-0067/19/3/858/s1.
