# Supplementary figures and images for: Transcriptomic Profiles of Brain Provide Insights into Molecular Mechanism of Feed Conversion Efficiency in Crucian Carp (Carassius auratus)
Source: Int J Mol Sci. 2018 Mar 14;19(3):858. doi: 10.3390/ijms19030858 (PMC5877719; doi:10.3390/ijms19030858)

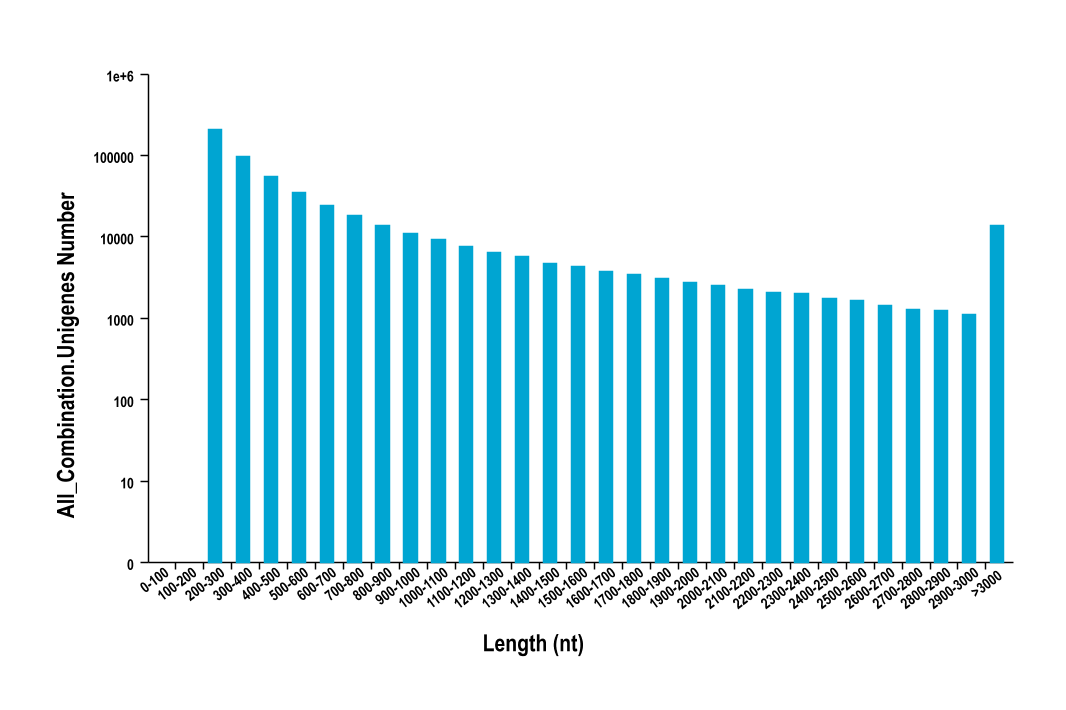

Supplement: Supplementary file 1 [file ijms-19-00858-s001.zip › Figure S1.png]
